# The Mediating Role of Job Satisfaction in the Relationship Between Perceived Organizational Support and Nurses’ Turnover Intentions

**DOI:** 10.1155/jonm/2960879

**Published:** 2026-07-22

**Authors:** Wejdan Shaqiqi

**Affiliations:** ^1^ College of Nursing, King Saud Bin Abdulaziz University for Health Sciences, Riyadh, Saudi Arabia, ksau-hs.edu.sa; ^2^ King Abdullah International Medical Research Center, Riyadh, Saudi Arabia, kaimrc.med.sa; ^3^ Ministry of the National Guard - Health Affairs, Riyadh, Saudi Arabia

**Keywords:** healthcare, intent to leave, mediation, nurses, retention, satisfaction, Saudi Arabia, stay, turnover

## Abstract

**Background:**

Investigating the factors that influence nurses’ turnover intention (TI) is crucial for maintaining a sustainable and efficient nursing workforce. While previous studies have explored the relationship between TI, perceived organizational support (POS), and job satisfaction (JS), few have examined the mediating effect of JS in this relationship.

**Aim:**

To assess nurses’ TI and its relationship with POS and JS, as well as the mediating role of JS.

**Methods:**

A cross‐sectional, descriptive, correlational research design was used. A convenience sample of 141 nurses was recruited from two hospitals in Saudi Arabia. Data were collected using a web‐based survey that included the Perceived Organizational Support Survey and the Michigan Organizational Assessment Questionnaire Job Satisfaction and Turnover Intention Subscales.

**Results:**

The participants had moderate TI (*M* = 10.22, SD ± 3.41), with 46.1% reporting a high level. TI was negatively correlated with POS (*r* = −0.393, *p* < 0.001) and JS (*r* = −0.453, *p* < 0.001). Both variables significantly predicted TI (*F* [2.138] = 21.98, *R* ^2^ = 0.231, *p* < 0.001), explaining a moderate proportion of its variance. A bootstrap test showed that JS had a partial mediating effect on the impact of POS on TI (*B* = − 0.09, SE = 0.03, 95% bootstrap CI [−0.14, −0.04]). The proportion mediated was 0.45, *p* *< *0.001, indicating that approximately 45% of the total effect of POS on TI operated indirectly through JS.

**Conclusion:**

Nurse leaders and policymakers should prioritize enhancing nurses’ JS as a critical strategy in nursing retention, as it may not only directly reduce TI but also indirectly strengthen the effect of POS on TI.

## 1. Introduction

Nurses are the backbone of the healthcare sector and represent a significant proportion of the healthcare workforce. They are responsible for continuous patient monitoring, delivering treatment plans, and ensuring safe, high‐quality care [1]. An adequate and sustainable nursing workforce is essential for population health, especially with the aging population and the burden of noncommunicable diseases [2]. Nurses are at the forefront of patient care and play a significant role in emergencies and crises, such as outbreaks and pandemics [2]. Nevertheless, nurse shortages present a global and local challenge. According to the WHO [2], despite the increasing number of nurses globally, inequities remain, with an estimated shortage of 5.8 million nurses. The distribution of nurses also varies substantially across regions (for example, African and Eastern Mediterranean regions have fewer nurses) and between high‐income and low‐income countries [2].

Nurse turnover and career changes pose additional threats to nursing resources, reduce patient care and satisfaction, increase the workload on other nurses, and create high costs for healthcare systems [3]. High nurse turnover is a global challenge that disrupts the quality of patient care and organizational performance. It increases recruitment and training costs, exacerbates staffing shortages, and adversely affects healthcare quality and patient safety. According to a meta‐analysis, the turnover rate among 213,314 nurses from 14 countries ranged from 8% to 36.6%, with a global rate of 16% [4]. A report from the Saudi Health Council showed a high nurse turnover rate of 20% in 2019, leading to an estimated annual cost of USD 693.33 million to the Saudi health system [5].

Turnover intention (TI), the final stage of withdrawal cognition, is defined as an employee thinking about quitting and forming the intention to seek alternative employment [6]. Nurses who leave their jobs often hold this intention for some time before resigning, with the final decision influenced by multiple personal and institutional factors [7]. A study found that 22 of 112 nurses with TI left their jobs within a year [8]. Given that TI is a strong predictor of actual turnover, understanding nurses’ TI and its influencing factors is a key step in improving nurse retention [9].

At the institutional level, perceived organizational support (POS) plays an important role both in contributing to and buffering against TI [10]. POS refers to employees’ perception of whether the organization values their contributions and cares about their well‐being [11]. When nurses believe that organizational support is high, they are less likely to leave. They also demonstrate greater job commitment and improved performance, motivated by a desire to contribute to the organization [12]. Another significant factor is job satisfaction (JS), which involves many aspects of both the organization and the job, including work environment, professional relationships, and rewards [13]. Nurses who experience high JS are more likely to remain in their positions, contributing to better patient outcomes and more stable healthcare systems [14]. Conversely, low JS often leads to TI, where nurses consider leaving their roles or the profession altogether [14].

Given the global impact of nurse turnover, this study aims to investigate TI among nurses in Saudi Arabia and its association with POS and JS. Continuous updates on the prevalence of TI are essential for understanding its current magnitude and informing strategies to address the issue. Additionally, Saudi Arabia is a country where most of the nursing workforce is exported [5], which also provides a global perspective. While literature supports that POS and JS are predictors of TI, empirical studies in the Saudi context remain limited. Existing studies indicate that TI is prevalent among nurses in Saudi Arabia [15, 16]; However, research examining the factors contributing to this phenomenon is relatively scarce compared with the extensive body of evidence from Western and East Asian contexts. Healthcare organizations in Saudi Arabia differ from those in many other countries due to the country’s rapidly evolving healthcare system, workforce composition, and ongoing national healthcare reforms under Saudi Vision 2030. Unlike many Western healthcare systems that rely primarily on locally trained nurses, Saudi healthcare organizations depend on expatriate nurses from other countries [5]. This multicultural workforce creates a complex organizational environment characterized by cultural diversity, communication challenges, varying professional expectations, and adaptation difficulties, all of which can influence nurses’ perceptions of organizational support and workplace satisfaction.

The healthcare system in Saudi Arabia continues to face significant nursing shortages and high turnover rates, particularly among expatriate nurses due to challenges related to cultural adjustment [7]. These contextual differences make the study particularly important within Saudi healthcare organizations. While TI is often linked primarily to salary, workload, or career advancement opportunities [7], in the Saudi context, organizational support may play a broader and more critical role because nurses often depend on their organizations not only for professional support but also for social integration, accommodation, cultural adaptation, and psychological well‐being [7]. Therefore, further research is needed to examine these relationships within the Saudi cultural and organizational healthcare institutions.

Although previous studies have established POS and JS as important predictors of TI, limited research has examined the mechanisms through which POS influences TI. In particular, few studies have investigated the mediating role of JS in the relationship between POS and TI [12, 17]. This study aims to address the gap in the existing literature by investigating POS, JS, and TI among nurses in Saudi Arabia and explores whether JS mediates the association between POS and TI among nurses. Examining these relationships is essential for understanding the factors that influence nurses′ intentions to leave their jobs and for informing the development of effective retention strategies within the healthcare sector.

### 1.1. Aim

This study aimed to (1) assess nurses’ TI levels and its mean differences according to level of POS and JS; (2) examine the correlation among TI, POS, and JS; (3) assess the effects of POS and JS on TI; and (4) investigate the mediating role of JS in the relationship between POS and TI.

### 1.2. Theoretical Framework

This study and its hypotheses are grounded in Social Exchange Theory (SET), a prominent paradigm for understanding employee behavior within organizations [18]. Central to this theory is the norm of reciprocity, which proposes that individuals engage in reciprocal relationships based on perceived support and benefits. Within this framework, positive organizational actions trigger a sense of obligation that influences employee attitudes and behaviors.

In this context, POS is expected to enhance nurses’ levels of JS. Nurses who feel supported by their organization are more likely to experience positive emotions toward their work, perceive fairness in organizational practices, and feel valued. Consequently, these positive experiences increase their satisfaction.

JS serves as a critical internal psychological state that translates organizational inputs into behavioral outcomes. Based on SET, when the “rewards” provided by the organization, in the form of POS, meet or exceed a nurse’s expectations, it leads to a state of high JS. This satisfaction acts as a mediator; it is the evaluative response to the organization’s supportive actions, which subsequently influences the nurse’s intent to maintain the professional relationship, making them less likely to consider leaving the organization.

Therefore, this study proposes that JS mediates the relationship between POS and TI. Specifically, when nurses perceive high organizational support, they reciprocate through increased satisfaction, which subsequently decreases their intention to leave the organization.

### 1.3. Hypotheses

• H1: POS has a negative effect on TI.
• H2: JS has a negative effect on TI.
• H3: JS mediates the relationship between POS and TI.

## 2. Methods

### 2.1. Study Design

A cross‐sectional, correlational design was applied to accomplish the research aims. The data were collected from two hospitals in two different main cities in Saudi Arabia (Jeddah and Riyadh) at one time point using a web‐based survey. The study adhered to the Strengthening the Reporting of Observational Studies in Epidemiology (STROBE) guidelines.

### 2.2. Setting

The study was conducted at two hospitals: a public hospital in Jeddah and a military hospital in Riyadh. These hospitals were selected because they are located in the two largest cities in Saudi Arabia and are large hospitals that provide primary, secondary, and tertiary health care services, with more than 1000 nurses across different departments. Selecting large hospitals operating under different healthcare systems and organizational policies allows for better identification of variations in organizational support practices and their impact on nurses’ JS and TI. In addition, the inclusion of hospitals with a significant number of nurses helped ensure a sufficiently diverse sample, thereby enhancing the quality of statistical analysis and improving the generalizability of the study findings.

### 2.3. Sample, Sampling, and Participants

A convenience sampling method was used to recruit nurses who were willing to participate and had at least 6 months of work experience in the institution. Nursing students and interns were excluded from the study. To enhance sample homogeneity, data were obtained from nurses employed in the same clinical units across both hospitals, namely, surgical wards, medical wards, intensive care units, and emergency departments. Nurses assigned to pediatric, maternity, oncology, and psychiatric departments were excluded to maintain consistency between the two hospitals and to minimize variability related to unit‐specific organizational conditions.

G∗Power 3.1.94 software was used to determine the minimum sample size required to run the analysis in this study. With a significance level of 0.05, power of 0.80, an effect size of 0.32 based on previous studies [12, 19], and two predictors, the minimum sample size was 111 participants. A total of 153 participants completed questionnaires. Following data screening, 12 questionnaires were excluded due to substantial missing data on key study variables, resulting in a final analytical sample of 141 participants.

### 2.4. Data Collection

A web‐based survey created in Google Forms was used to recruit nurses. The survey was administered in English, as it is the official working language within the healthcare system in Saudi Arabia. After obtaining ethical approval, the nursing directors at both hospitals were approached to explain the aim and nature of the study, and their permission was obtained to collect data. Due to the sensitivity of the topic, data were collected through email to ensure confidentiality and encourage honest responses. An email was sent to the institutional email addresses of nurses in the targeted departments. It included a recruitment statement, the web‐based survey link, and the researcher’s contact information for inquiries. To increase the response rate, a reminder was sent 2 weeks later. Data collection began in September 2024 and ended in November 2024, after the target sample size was reached.

### 2.5. Measurement

Data were collected using a questionnaire consisting of three parts. The first part obtained participants’ demographic information, including gender, age, qualification, position, and years of nursing experience. The second part was the eight‐item Survey of POS [11], which measures employees’ general belief that their organization values their membership and is concerned about their well‐being using a seven‐point Likert scale ranging from 1 (*strongly disagree*) to 7 (*strongly agree*). A sample item is as follows: the organization really cares about my well‐being. Total scores ranged from 8 to 56 and were divided into three equal categories: low (*M* ≤ 24), moderate (*M* = 25 – 40), and high (*M* ≥ 41), with higher scores indicating higher levels of POS. The scale has high validity and reliability [11], and the internal reliability in this study was *α* = 0.778. The third part was the JS and TI Subscales of the Michigan Organizational Assessment Questionnaire [20], comprising three items in each subscale, scored using a five‐point Likert scale ranging from 1 (*strongly disagree*) to 5 (*strongly agree*). A sample item is as follows: I will probably be looking for a new job in the next year. Total scores range from 3 to 15 and are divided into three equal categories: low (*M* ≤ 6), moderate (*M* = 7–10), and high (*M* ≥ 11), with higher scores indicating higher levels of the construct. The scales have satisfactory reported validity and reliability, and the internal consistency of the subscales in this study was *α* = 0.71 and 0.87, respectively.

### 2.6. Data Analysis

Statistical analyses were conducted using the Statistical Package for the Social Sciences (SPSS) Version 30. Prior analyses, the assumptions of linearity, normality, homoscedasticity, independence of errors, and multicollinearity were assessed. Visual inspection of scatterplots and residual plots supported the assumptions of linearity and homoscedasticity. The histogram and normal probability plot indicated that residuals were approximately normally distributed. The Durbin–Watson statistic was within the acceptable range, indicating independence of errors. Multicollinearity was not evident, as tolerance values exceeded 0.20 (0.744) and VIF values were below 5 (1.34).

Descriptive statistics, including frequencies, percentages, means, standard deviations, and ranges, were used to summarize demographic variables and the scores for TI, POS, and JS. Bivariate correlation analyses examined the relationships among TI, POS, and JS. Multiple linear regression was used to determine whether POS and JS significantly predicted TI. Bootstrapping procedures with 5000 resamples were conducted using the SPSS PROCESS Macro Model No. 4 to investigate the mediating role of JS in the relationship between POS and TI [21]. This approach is appropriate for regression‐based mediation models using observed composite variables and provides robust estimates of indirect effects. Unlike traditional mediation approaches, using the PROCESS macro with bootstrapping analysis strengthens mediation testing. PROCESS directly estimates the mediation pathway and determines whether the indirect effect is statistically significant. Bootstrapping improves this process by repeatedly resampling the data, typically 5000 times, to generate confidence intervals without assuming normal distribution of the indirect effect. This increases the production of more robust estimates of mediation effects, particularly in behavioral and nursing research where data are often nonnormally distributed and relationships among variables are complex (Hayes, 2018). Finally, differences in TI, POS, and JS by demographic variables were analyzed using one‐way ANOVA and independent‐samples *t* tests. Statistical significance was set at *p* < 0.05.

### 2.7. Ethical Considerations

Approval for this study was obtained from the Institutional Review Board of King Abdullah International Medical Research Center (KAIMRC) and the nursing directors of the participating hospitals (IRB/0261/23). The study was conducted in accordance with the principles of the Declaration of Helsinki and the Belmont Report. Participants received a recruitment statement explaining the purpose of the study and its potential risks and benefits. The statement also informed participants of their rights, including voluntary participation, the right to refuse to participate, and the right to withdraw from the study at any time without consequences. To ensure privacy and confidentiality, no personal identifiers were collected. Electronic tracking, including IP address recording, was disabled to allow for anonymous responses. Data were accessed only by the researcher and reported in an aggregate form. Participants indicated their consent to participate by completing the consent form displayed on the first page of the survey.

## 3. Results

### 3.1. Demographic Characteristics

Table 1 presents the participants’ demographic characteristics. The mean age of participants was 35.66 (SD ± 7.65), and the majority were female (85.7%) and non‐Saudi (63.1%). Most participants held a bachelor’s degree (63.8%), were staff nurses (66.9%), and had more than 6 years of experience in nursing (37.9%). More than half of the participants were from the military hospital in Riyadh (57.9%).

**TABLE 1:** Demographic characteristics of the participants (*N* = 141).

Variable	*N* (%) or M (SD)
Age	35.66 (7.65)
Gender^∗^	
Female	120 (85.7)
Male	20 (14.3)
Nationality	
Saudi	52 (36.9)
Non‐Saudi	89 (63.1)
Marital statues	
Married	70 (49.6)
Single	65 (46.1)
Divorced/widowed	6 (4.3)
Educational level	
Diploma	34 (24.1)
Bachelors	90 (63.8)
Master or higher	17 (12.1)
Position^∗^	
Staff nurse	93 (66.9)
Charge nurse	32 (23.0)
Head nurse/assistant head nurse	14 (10.1)
Years of experience in nursing^∗^	
Less than a year	24 (17.1)
1–3 years	41 (29.3)
4–6 years	22 (15.7)
More than 6 years	53 (37.9)
Hospital^∗^	
Public in Jeddah	59 (42.1)
Military in Riyadh	81 (57.9)

^∗^Frequencies may not equal the total sample size due to missing responses.

### 3.2. TI Level and its Mean Difference According to POS and JS Levels

Table 2 displays the mean TI, POS, and JS scores. The average total TI score was moderate (*M* = 10.22, SD ± 3.41), with almost half of the participants reporting a high intention to leave their job within a year (46.1%). POS scores were low (*M* = 22.5, SD ± 6.56), with more than half of the participants reporting low POS (58.9%). JS scores were moderate (*M* = 10.19, SD ± 2.54), with about half of the participants reporting high satisfaction (48.9%).

**TABLE 2:** Turnover intention scores and its mean differences by levels of perceived organizational support and job satisfaction (*N* = 141).

Variables	Turnover intention (*M* = 10.22, SD ± 3.41)	Perceived organizational support (*M* = 22.95, SD ± 6.56)				Job satisfaction (*M* = 10.19, SD ± 2.54)			
	*N* (%)	*N* (%)	M (SD)	*t*	*p*	*N* (%)	M (SD)	*F*	*p*
Low	20 (14.2%)	83 (58.9)	11.06 (3.23)	3.61	< 0.001^∗∗∗^	10 (7.1%)	12.50 (3.92)	12.94	< 0.001^∗∗∗^
Moderate	56 (39.7%)	58 (41.1)	9.03 (3.33)			62 (44.0%)	11.33 (2.96)		
High	65 (46.1%)	—	—			69 (48.9%)	8.89 (3.22)		

^∗∗∗^ *p* < 0.001.

For POS, the results revealed a statistically significant difference in TI across the three groups (*t* [139] = 3.59, *p* < 0.001, Cohen’s *d* = 0.62), indicating a medium effect size. Nurses with moderate POS (*M* = 9.03, SD ± 3.3, *p* < 0.001) demonstrated significantly lower TI than those with low POS (*M* = 11.06, SD ± 3.23). Similarly, JS levels showed a statistically significant difference in TI (*F* [2.138] = 12.94, *p* < 0.001, *η* ^2^ = 0.15), indicating a medium‐to‐large effect size. Participants with high JS (*M* = 8.89, SD ± 3.22, *p* < 0.001) reported less TI than those with moderate (*M* = 11.33, SD ± 2.96) and low (*M* = 12.50, SD ± 3.92) JS. These findings indicate that higher POS and JS levels are associated with reduced TI among nurses.

### 3.3. TI Relationship With POS and JS

Pearson’s product–moment correlation was used to determine the association among TI, POS, and JS (Table 3). TI was negatively and moderately correlated with POS (*r* = −0.393, *p* < 0.001) and JS (*r* = −0.453, *p* < 0.001). Furthermore, a moderate positive correlation was observed between POS and JS (*r* = 0.506, *p* < 0.001).

**TABLE 3:** Correlation between turnover intention, perceived organizational support, and job satisfaction.

Variables	1	2	3
1. Turnover intention	1		
2. Perceived organizational support	−0.393^∗∗∗^	1	
3. Job satisfaction	−0.453^∗∗∗^	0.506^∗∗∗^	1

*Note:* Correlation is significant at the 0.01 level (two‐tailed).

^∗∗∗^ *p* < .001.

Multiple linear regression analysis examined whether POS and JS influenced TI (Table 4). The findings indicated a significant relationship (*F* [2.138] = 21.98, *R* ^2^ = 0.231, *p* < 0.001). POS (*β* = −0.220, *p* = 0.011) and JS (*β* = −0.342, *p* < 0.001) significantly and negatively predicted TI. The model demonstrated a medium‐to‐large effect size (*f* ^2^ *= *0.30) according to Cohen’s criteria. Nurses with higher POS and JS had lower TI.

**TABLE 4:** Influence of perceived organizational support and job satisfaction on turnover intention (*N* = 140).

Predictors	Turnover intention						
	B^a^	SE B	β^b^	*t*	*p*	95% CI	
						Lower	Upper
Perceived organizational support	−0.115	0.045	−0.220	−2.566	0.011^∗^	−0.203	−0.026
Job satisfaction	−0.458	0.115	−0.342	−3.978	< 0.001^∗∗∗^	−0.685	−0.230
Model summary	(*F* [2. 138] = 21.98, *R* ^2^ = 0.231, *p* < 0.001)						

*Note:* A multiple linear regression model was used; R^2^ = adjusted R‐squared value; B^a^ = coefficient; β^b=^ Beta standardized coefficient.

^∗^ *p* < .05.

^∗∗∗^ *p* < .001.

### 3.4. The Mediation Effect of JS on the Relationship Between POS and TI

**FIGURE 1:**
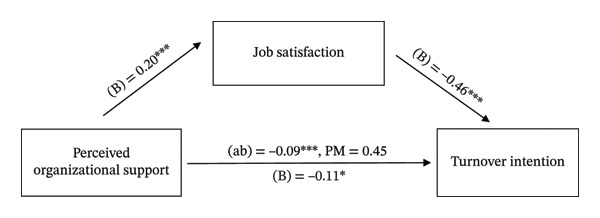
Regression of mediating effect of job satisfaction on the relationship between perceived organizational support and turnover intention.

The mediating role of JS in the relationship between POS and TI is presented in Table 5 and Figure 1. The findings indicated that POS had a direct effect on TI (*B* = −0.11, *p = *0.0, 95% CI [−0.20, −0.03]). Moreover, POS had a significant effect on JS (*B* = 0.20, *p* *< *0.001, 95% CI [0.14, 0.25]), and JS had a significant effect on TI (*B* = −0.46, *p* *< *0.001), within the 95% CI [−0.−0.69, −0.23]. POS had a significant effect on TI of *B* = −0.09, within the 95% CI [−0.14, −0.04], through JS. The proportion of mediation, representing the ratio of the indirect effect to the total effect, was P_M_ = 0.45, indicating that 45% of the total effect of POS on TI occurred through JS. The results revealed a significant total effect (*B* = −0.20, SE = 0.04, *t* = −5.04, *p* *< *0.001, 95% CI [−0.28, −0.12]). This indicates that nurses with greater POS reported lower TI overall.

**TABLE 5:** Regression of mediating effect of job satisfaction by bootstrapping (*N* = 141).

Effect	Variables	B	SE	*t*	*p*	95% CI		P_M_
						LLCI	ULCI	
Direct effect	Perceived organizational support ⟶ turnover intention (c′)	−0.11	0.04	−2.57	0.01^∗^	−0.20	−0.03	0.45
Indirect effect	Perceived organizational support ⟶ job satisfaction (a)	0.20	0.03	6.91	0.00^∗∗∗^	0.14	0.25	
Indirect effect	Job satisfaction ⟶ turnover intention (b)	−0.46	0.12	−3.98	0.00^∗∗∗^	−0.69	−0.23	
Indirect effect	Perceived organizational support ⟶ job satisfaction ⟶ turnover intention (ab)	−0.09	0.03			−0.14	−0.04	
**Total effect**	(c′ + ab)	−0.20	0.04	−5.04	0.00^∗∗∗^	−0.28	−0.12	

*Note:* 5000 bootstrapping were re‐extracted; B = B‐coefficient, SE = standard error, CI = confidence interval, LLCI = lower limit of B in 95% confidence interval, ULCI = upper limit of B in 95% confidence interval, P_M_ = proportion mediated, ratio between indirect effect and total effect.

^∗^ *p* < 0.05.

^∗∗∗^ *p* < 0.001.

Mean difference of TI, POS, and JS according to demographic characteristics.

Independent‐samples *t* tests and one‐way ANOVA were used to assess mean differences in nurses’ POS and JS on TI according to demographic factors (Table 6). The results revealed that Saudi nurses had lower POS (*M* = 21.59, SD ± 6.41, *p* = 0.031) and JS (*M* = 9.79, SD ± 2.97, *p* = 0.032) and higher TI (*M* = 11.03, SD ± 3.48, *p* = 0.015) than expatriate nurses. Participants with a master’s degree or higher had higher TI (*M* = 11.82, SD ± 2.35, *p* = 0.039) than those with a lower degree. Staff nurses had a higher POS (*M* = 23.64, SD ± 6.19, *p* = 0.040) and less TI (*M* = 9.77, SD ± 3.47, *p* = 0.014) than charge, assistant, and head nurses. Participants from the military hospital had higher POS (*M* = 23.86, SD ± 6.80, *p* = 0.033) and JS (*M* = 10.60, SD ± 2.32, *p* = 0.016) and lower TI (*M* = 9.65, SD ± 3.11, *p* = 0.014) than those from the public hospital. Lastly, participants with 4 or more years of experience had lower POS (*M* = 21.97, SD ± 6.39, *p* = 0.028) but higher JS (*M* = 10.60, SD ± 2.31, *p* = 0.018) than those with less than 4 years of experience.

**TABLE 6:** Mean differences in perceived organizational support, job satisfaction, and turnover intention according to the demographic factors (*N* = 141).

Demographic factors	Categories	*N*	Perceived organizational support				Job satisfaction				Turnover intention			
			M	(SD)	*t* ‐ *F*	*p*	M	(SD)	*t* ‐ *F*	*p*	M	(SD)	*t* ‐ *F*	*p*
Nationality	Local	52	21.59	6.41	1.888	0.031^∗^	9.67	2.79	−1.862	0.032^∗^	11.03	3.48	2.187	0.015^∗^
	Expatriate	89	23.74	6.561			10.49	2.35			9.75	3.29		

Educational level	Diploma	34	24.02	5.51	0.688	0.504	10.41	2.65	0.606	0.547	9.26	3.33	3.334	0.039^∗^
	Bachelors	90	22.72	6.59			10.22	2.51			10.28	3.51		
	Master or higher	17	22.00	8.31			9.58	2.57			11.82	2.35		

Position	Staff Nurse	93	23.64	6.19	1.761	0.040^∗^	10.20	2.59	0.083	0.467	9.77	3.47	2.223	0.014^∗^
	Other	48	21.60	7.11			10.16	2.48			11.10	3.13		

Hospital	Public	59	21.79	6.09	1.854	0.033^∗^	9.67	2.74	2.157	0.016^∗^	10.96	3.68	2.218	0.014^∗^
	Military	81	23.86	6.80			10.60	2.32			9.65	3.11		

Experience in nursing	3 years or less	65	24.09	6.63	1.928	0.028^∗^	9.70	2.73	2.110	0.018^∗^	9.92	3.48	0.978	0.165
	4 years or more	76	21.97	6.39			10.60	2.31			10.48	3.34		

*Note:* *p* value is calculated using an independent sample *t*‐test or a one‐way ANOVA.

^∗^ *p* < .05.

## 4. Discussion

This study assessed the TI and its association with POS and JS. It also investigated the mediating role of JS in the relationship between POS and TI. The overall level of TI within a year was moderate, with nearly half of the participants reporting a high TI (46.0%). This finding is similar to the results from a review of nurses’ TI in Saudi Arabia [22]. This percentage is concerning because it indicates a significant risk of workforce instability, and it highlights the urgent need for targeted retention strategies in healthcare settings. TI varied by participants’ levels of POS and JS. Participants who reported higher levels of POS and JS exhibited significantly lower TI. In line with the literature, TI was negatively correlated with, and predicted by, both POS and JS [12, 14, 23]. POS and JS accounted for 23.1% of the variance in TI. This proportion highlights the role of both variables as critical determinants of TI. These findings indicate that actively fostering supportive environments and enhancing JS might effectively reduce nurses’ intention to leave, ultimately leading to improved healthcare outcomes and enhancing nursing retention, which is vital for the overall functioning of healthcare systems [3].

POS and JS were positively correlated, indicating that enhancing organizational support can improve JS. This association has also been reported in previous studies [23, 24]. When nurses feel supported through a positive work environment, effective management, adequate resources, and opportunities for professional development, they are more likely to experience higher JS and less likely to consider leaving their job or the profession [25].

JS served as a mediator between POS and TI, accounting for 45% of the total effect of POS on TI. This percentage is slightly lower than that reported in a similar study conducted in Iran (63.9%) [17]. Another study also found that JS mediated the association between POS and nurses’ intention to stay by 50% [24]. The variation in percentages may be explained by differences in cultural and organizational contexts, as well as the characteristics of the participants. JS was also identified as a mediator between several factors associated with nurses’ TI, such as career identity [26], hope [26], burnout [27], work stress [28], workplace violence [29], and career compromise [30]**.** This highlights its key role as a direct determinant of TI and an indirect factor through various personal, professional, and organizational variables. Therefore, organizations should prioritize enhancing nurses’ satisfaction to improve nursing retention.

These findings are consistent with and support SET demonstrating the relationships among POS, JS, and TI. In addition, they extend this theoretical framework in the context of Saudi Arabia by confirming JS as a partial mediator between POS and TI. The mediation results indicate that JS partially explains the relationship between POS and TI. Specifically, while a substantial indirect effect is observed through JS, a meaningful direct effect of POS on TI remains. This can be explained by the theory that nurses may reciprocate to the organization directly through commitment, in addition to satisfaction. Moreover, although JS plays an important mediating role, the persistence of a direct effect of POS on TI further suggests that other underlying mechanisms may be involved, highlighting the need for future research to explore additional explanatory pathways.

We observed variations in the mean scores of the study variables based on personal and professional factors. Specifically, Saudi nurses reported higher TI and lower POS and JS than their non‐Saudi counterparts. These differences may be related to contextual factors such as work environment, cultural expectations, career development opportunities, and organizational policies, which can significantly influence nurses’ perceptions and attitudes [25]. Nurses with higher educational qualifications reported higher TI, possibly due to greater expectations for career advancement and duties, professional autonomy, and involvement in decision‐making; when these expectations are unmet, dissatisfaction and a stronger desire to leave may result [31, 32]. Conversely, nurses with more experience reported lower POS but higher JS. Our findings contrast with a study of emergency room nurses in Iran, which found a positive correlation between years of experience and POS [33]. This contrast highlights the complexity of nurses’ professional journey: while experienced nurses may develop coping mechanisms and intrinsic satisfaction from their roles, they might still feel undervalued or unsupported by their institutions [14]. Interestingly, nurses in leadership positions also felt less supported and exhibited higher TI, despite promotion opportunities, suggesting that leadership roles may involve greater stress and responsibility. With limited access to structural support, these demands may outweigh the perceived benefits of career progression [7]. Furthermore, observed differences between public and military hospitals underscore the influence of institutional policies and support systems. Variations in leadership styles, resource allocation, and organizational culture likely contribute to these disparities, emphasizing the need for tailored strategies to enhance support and retention across different healthcare settings [12]. Nevertheless, these findings should be interpreted cautiously due to the relatively small sample size and the unequal distribution of participants across groups.

### 4.1. Implications

This research provides a foundation for designing effective interventions to enhance nurse retention and improve the quality of healthcare delivery. Based on the findings of the current study, POS and JS were significant predictors of nurses’ TI. Therefore, strengthening POS and JS through targeted managerial strategies is essential to increase nurses’ intention to stay. Previous studies suggest that improving the work environment is central to boosting both POS and JS, which can be achieved by ensuring adequate staffing levels; fostering a safe, positive, and respectful workplace culture; promoting nurses’ autonomy and involvement in decision‐making; and implementing mentoring programs [25, 34]. Healthcare leaders should aim to increase both POS and JS by adopting transformational leadership practices that emphasize supportive supervision, open and transparent communication, effective conflict resolution, and fair workload delegation [25].

More importantly, enhancing JS is likely to yield the quickest results in addressing TI, as it has both direct and indirect effects on TI. This can be achieved by supporting nurses’ well‐being, enhancing recognition and appreciation, and establishing team‐building and recreational activities at both the unit and organizational levels [25, 34]. Offering competitive salaries and benefits, including retention bonuses, and implementing fair and transparent promotion and pay raise policies are critical for JS [25]. At the individual level, providing opportunities for professional and career development, such as stress management courses and leadership training, can also improve JS, reduce TI, and support long‐term nurse engagement [25, 34].

### 4.2. Limitations and Recommendations for Future Research

This study has several limitations. It employed a cross‐sectional design, which limits the ability to establish causal relationships between the study variables. Additionally, the 141 participants from only 2 hospitals (public and military) in 2 major cities may reduce the generalizability of the findings to the broader nursing population, particularly nurses working in private hospitals, rural hospitals, or other regions of Saudi Arabia. Convenience sampling and self‐reported data cause several potential biases that can affect the accuracy of the findings, including self‐selection bias, recall bias, response bias, and social desirability bias.

Based on the findings and limitations of the present study, several recommendations are proposed for future research. First, future research should build on these findings by considering using multiple data collection methods rather than relying solely on self‐reported questionnaire data. Qualitative interviews, field observations, and organizational performance records and a longitudinal study may provide richer insights and reduce potential biases associated with self‐report measures, including social desirability bias and recall bias. The use of mixed‐method approaches may, therefore, enhance the validity and reliability of research findings.

Future studies may analyze differences between groups to gain a deeper understanding of contextual and demographic influences on TI. Comparative studies between public and private hospitals, urban and rural healthcare settings, or healthcare institutions in different geographic regions may reveal important variations in organizational practices and employee experiences. In addition, comparisons based on nurses’ years of experience, specialization, age, or educational background may provide more detailed insights into factors affecting TI among different employee groups.

Future studies are encouraged to incorporate additional mediating or moderating variables that may influence the POS, JS, and TI. Variables such as psychological stress, organizational culture, work–life balance, leadership style, and personal or social factors may provide a more comprehensive understanding of the complex mechanisms underlying employee turnover behavior. Incorporating such variables may help explain how and under what conditions organizational support influences employees’ attitudes and intentions. Lastly, future research is encouraged to employ more advanced analytical techniques, such as structural equation modeling (SEM), moderation analysis, and mediation analysis, to examine direct and indirect relationships among variables more accurately.

## 5. Conclusion

This study contributes to the growing body of literature by providing empirical evidence on nurses’ TI level, the influence of both POS and JS on nurses’ TI, and the mediating role of JS in strengthening the effect of organizational support on these intentions. The results indicated that almost half of nurses have a high TI. Moreover, POS and JS jointly explained 23.1% of the variance in TI and both were negatively associated with TI. JS acts as a mediating variable, accounting for 45% of the total impact of POS on TI. The research results thereby confirm all proposed hypotheses and support the SET, which explains that POS leads nurses to reciprocate with positive attitudes to JS and reduces their TI. These findings underscore the need to improve nursing workforce retention and highlight the importance of comprehensive policies and practices aimed at enhancing both POS and JS. By fostering a supportive work environment and leadership, organizations may reduce nurses’ TIs and improve retention rates, positively affecting both organizational performance and patient care.

## Funding

No funding was received for this research.

## Ethics Statement

This study is in accordance with the Declaration of Helsinki (1964) and was approved by the Institutional Review Board of King Abdullah International Medical Research Centre (KAIMRC).

## Conflicts of Interest

The author declares no conflicts of interest.

## Data Availability

The data that support the findings of this study are available on request from the corresponding author. The data are not publicly available due to privacy or ethical restrictions.
